# Identification of selected genetic polymorphisms in polycystic ovary syndrome in Sri Lankan women using low cost genotyping techniques

**DOI:** 10.1371/journal.pone.0209830

**Published:** 2018-12-31

**Authors:** Umayal Branavan, Kajan Muneeswaran, Sulochana Wijesundera, Surangi Jayakody, Vishvanath Chandrasekharan, Chandrika Wijeyaratne

**Affiliations:** 1 Department of Obstetrics and Gynecology, Faculty of Medicine, University of Colombo, Colombo, Sri Lanka; 2 Department of Chemistry, Faculty of Science, University of Colombo, Colombo, Sri Lanka; 3 Department of Psychiatry, Faculty of Medicine, University of Colombo, Colombo, Sri Lanka; 4 Department of Biochemistry and Molecular Biology, Faculty of Medicine, University of Colombo, Colombo, Sri Lanka; 5 Department of Community Medicine, Faculty of Medical Sciences, University of Sri Jayawardanapura, Nugegoda, Sri Lanka; Florida International University, UNITED STATES

## Abstract

**Background:**

Polycystic ovary syndrome (PCOS), the commonest endocrine disorder affecting young women, appears to be a multigenic trait with contributing genes being unclear. Hence, analysis of polymorphisms in multiple candidate genes is required. Currently available genotyping methods are expensive, time-consuming with limited analytical sensitivity.

**Aim:**

(i) Develop and validate high resolution melting (HRM) assay and allele-specific real-time quantitative PCR (AS-qPCR) for genotyping selected SNPs associated with PCOS.

(ii) Identify selected SNPs and their association with a Sri Lankan cohort of well-characterized PCOS.

**Methods:**

DNA was extracted from women with well-characterized PCOS from adolescence (n = 55) and ethnically matched controls (n = 110). FTO (Fat mass and obesity associated gene; rs9939609), FSHB (Follicle stimulating hormone beta subunit; rs6169), FSHR (Follicle stimulating hormone receptor; rs6165/rs6166), and INSR (Insulin receptor; rs1799817) genes were genotyped using HRM assay. GnRH1 (Gonadotropin releasing hormone; rs6185), LHB (Luteinizing hormone beta subunit; rs1800447/rs34349826) and LHCGR (Luteinizing hormone/choriogonadotropin receptor; rs2293275) genes were genotyped using AS-qPCR method. Genotyping results were validated using Sanger sequencing.

**Results:**

A significant association was observed within FTO gene polymorphism (rs9939609) and PCOS. Genotype frequency of FTO gene (rs9939609)—cases versus controls were TT-36.4% vs.65.4% (p<0.05), AT-23.6% vs.20.9%, AA-40% vs.13.6% (p<0.05). Genotype frequencies of the SNPs GnRH1 (rs6185), FSHB (rs6169), FSHR (rs6165 & rs6166), LHB (rs1800447 & rs34349826), LHCGR (rs2293275) and INSR (rs1799817) were not significantly different between cases and controls (p>0.05). Only the mutant alleles were observed for LHB rs1800447 and rs34349826 SNPs in both groups. The HRM and AS-qPCR assay results had 100% concordance with sequencing results.

**Conclusions:**

FTO gene rs9939609 polymorphism is significantly more prevalent among Sri Lankan PCOS subjects while the other selected SNPs of HPG axis genes and INSR gene showed no association. HRM and AS-qPCR assays provide a reliable, fast and user-friendly genotyping method facilitating wider implication in clinical practice.

## Introduction

Polycystic ovary syndrome (PCOS) is the commonest endocrine disorder affecting women of reproductive age with a prevalence varying between 5–13%. PCOS typically presents during adolescence with a wide spectrum of phenotypes that are characterized by any combination of anovulation (amenorrhoea, irregular cycles), androgen excess (hirsutism, acne, alopecia) and polycystic ovaries on ultrasound [1].

Based on current research there is strong evidence that genetic factors play an important role in the etiology of PCOS. Despite several studies dissecting the variants of genes from multiple biological pathways, the impact of the mode of inheritance of PCOS on its pathophysiology remains unclear [2]. PCOS appears to be a multigenic trait, although the contributing genes remain undefined [3,4,5,6]. Candidate genes of steroid hormone biosynthesis and metabolism, gonadotropin and gonadal hormones action, obesity and energy regulation, insulin secretion and action have been studied and implicated in the pathogenesis of PCOS. Some recent studies have identified varying SNP sites to be associated with PCOS in differing populations. Tian et al. reported an association of FSHB gene variant (rs11031010) with PCOS in Han Chinese women [7]. Wang et al. proposed RAD54B gene (involved in homologous recombination and repair of DNA) may contribute to the hyperandrogenism of PCOS in the Han Chinese population [8]. Moreover, Cui et al. showed a direct correlation between the genotypes and PCOS phenotype and concluded that every feature of PCOS has a specific genetic association linked to the aetiological pathway [9]. Additionally, the recent Genome-wide association study (GWAS) by Chen et al. showed 3 SNP sites on THADA, DENND1A and TOX3 to be associated with PCOS [10]. Brower et al. evaluated whether the variants associated with PCOS in Han Chinese are also associated with PCOS in white Europeans and concluded that DENND1A, THADA, FSHR, INSR and YAP1 loci are likely to play important roles in the etiology of PCOS across populations [11]. Nevertheless, Shim et al. carried out a pathway-based analysis of a GWAS dataset to elucidate which biological pathways were associated with genes in PCOS. The conclusion was that oocyte meiosis is the top-ranking biological pathway associated with PCOS [12].

It is noteworthy that the underlying pathway of the pathophysiology in each of the phenotypes of PCOS may differ, based on the genetic and environmental contributions. There may be a number of interlinking factors that affects the expression of PCOS. A single cause for PCOS is very unlikely [13]. Hence, multiple candidate genes associated with PCOS should be screened to determine its exact genetic basis.

Genotyping is now an important diagnostic means for elucidating most diseases. Among the advanced techniques available to study single nucleotide polymorphisms (SNPs), only a few are applicable for routine disease screening. Their applicability mainly depends on three factors: cost, time and accuracy.

The aims of the study were to (i) develop and validate high resolution melting (HRM) assay and allele-specific real-time quantitative PCR for genotyping selected SNPs associated with PCOS and (ii) identify selected SNPs and their association with PCOS in Sri Lankan women.

## Materials and methods

### Recruitment of subjects

This study was approved by the Ethics Review Committee, Faculty of Medicine, University of Colombo, Sri Lanka. Written informed consent was obtained from all participants. Consecutive well characterized cases were recruited from the Endocrine Clinic of the University Obstetrics and Gynaecology Department, Colombo. Diagnosis of PCOS was based on the Rotterdam criteria [14], with diagnostic certification made by a single clinical lead. The sample size required for consecutive subjects with well characterized PCOS manifesting from adolescence was 55; and by selecting double this number of controls, the sample size of control subjects was 110 (Schlesselman case control study formula was used for sample size calculation [15]).

#### Inclusion criteria

Inclusion criteria were women whose symptoms manifested from adolescent years (11–19 years WHO), with all 3 diagnostic criteria present from 16–19 years of age [16]. The lower limit of age selection was based on the mean age of menarche in Sri Lanka being 13 years and leaving an allowance of two additional years for regularization of menstruation [17].

Anovular PCOS or amenorrhoea/ oligomenorrhea: Anovular cycles are defined when the cycle length is more than 35 days, and the lack of demonstrable ovulation by mid cycle and luteal phase ultrasound scans, and mid-luteal serum progesterone [16]. Amenorrhoea—absence of menstrual periods for six months or more in a woman who has previously been menstruating. Oligomenorrhea—menstrual periods occurring at intervals of greater than 35 days, with only four to nine periods in a year.

Polycystic ovaries on ultrasound: defined by transvaginal or transabdominal ultrasound scan of ovaries, performed within the first 5 days from the onset of menstruation, and finding 24 or more follicles, measuring between 2 and 9 mm and/or an ovarian volume >10 cm^3^ [14].

Hyperandrogenism: Clinical evidence of hirsutism by modified Ferriman-Gallwey score (mFG) ≥8, serum testosterone (T) > 3.5nmol/L and/or free androgen index (FAI) >5 [16].

#### Exclusion criteria

Exclusion criteria included inherited disorders of IR such as Rabson–Mendenhall syndrome, Cushing syndrome, hyperprolactinaemia, untreated primary hypothyroidism, congenital adrenal hyperplasia or an androgen secreting ovarian/adrenal tumor; those taking corticosteroid, antiepileptic or antipsychotic drugs, history of hormonal contraception within the previous 6 months, pregnancy and the first postpartum year.

Control sample: Concurrently asymptomatic, normo-androgenic, normal cycling since adolescence, non-medicated, consenting women of reproductive age in whom PCOS was objectively excluded by clinical, biochemical and ultrasound assessment, were recruited as controls. The control subjects were recruited from a single work setting where health promotion programs were conducted from 2012 (3 years before the study). Working women of similar ethnic and social background as the affected subjects were invited to participate in the study.

### Clinical evaluation

Clinical evaluation was by a pre-tested questionnaire-based interview regarding: socio demographic factors, detailed menstrual and obstetric histories, infertility if relevant, the onset and degree of clinical symptoms of PCOS, drug history, family history of diabetes and other cardiovascular risk factors. Detailed physical examination included measurement of standing height to the closest centimeter [18] and weight in kilograms to calculate the BMI, waist and hip circumference and waist-to-hip ratio (WHR), resting blood pressure [19], hirsutism (FG score), frontal balding, distribution of acne and acanthosis nigricans [20].

Evaluation of the modified FG score was done by a single medically qualified clinical lead of the Department of Obstetrics and Gynecology, Faculty of Medicine, University of Colombo.

Ultrasound examinations were performed by a single trained medically qualified ultra sonographer under the supervision of the Radiology lead of the De Soysa Hospital for Women, Colombo.

### Biochemical and endocrine evaluation

Two milliliters venous blood was collected into plain sterile tubes from each subject and serum was extracted. Serum kisspeptin and testosterone levels were measured with ELISA kits (Phoenix Pharmaceuticals Inc., Belmond, CA and Teco Diagnostics, USA respectively) as per manufacture recommendation. Serum kisspeptin and testosterone concentrations were determined using the standard curve of known concentrations. Fasting blood glucose was measured in all subjects. Routine laboratory tests performed to diagnose/monitor PCOS (follicular phase FSH, LH, thyroid stimulating hormone, and fasting blood glucose/75 g oral glucose tolerance test) which were carried out at the quality controlled laboratory of the National Hospital Colombo were recorded for PCOS subjects.

### DNA extraction

DNA was extracted from blood samples (2ml) using genomic DNA extraction kit (Promega, USA), following the manufacturer’s protocol. The DNA samples were subsequently stored at −20°C.

### Selection for study of candidate genes and SNPs of PCOS

The selection of SNPs for this study was based on screening OMIM, SNPedia and ClinVar and the highly susceptible SNPs for PCOS reported from Asia were selected for the study.

**Fig 1:**
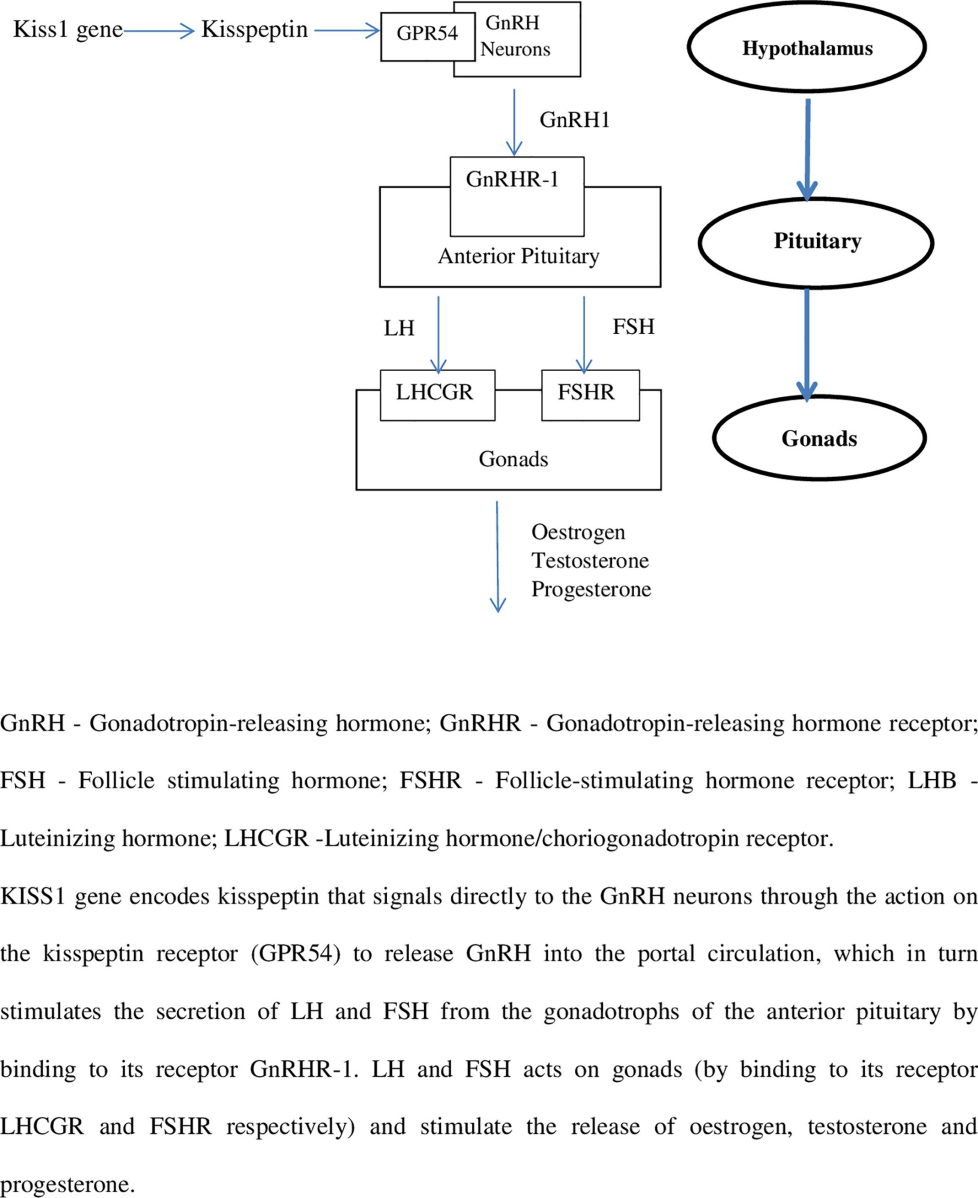
Diagrammatic representation of the HPG axis pathway in humans.

In view of deregulation of the hypothalamic pituitary gonadal (HPG) axis being linked to the pathophysiology of PCOS [21], we explored the potential for studying selected polymorphisms of genes associated with the HPG axis (Fig 1). The following HPG genes and their respective SNPs were selected: GnRH1 (rs6185), FSHB (rs6169), FSHR (rs616/rs6166), LHB (rs1800447/rs34349826) and LHCGR (rs2293275). Insulin resistance playing a central role in its pathophysiology with obesity and type 2 diabetes mellitus being common problems of PCOS [22], we also selected polymorphisms in the obesity associated gene (FTO—rs9939609) and Insulin receptor gene (INSR—rs1799817) that are reported to be more prevalent in Asians [23–25].

### Primer designing for high resolution melt (HRM) assay and allele-specific real-time quantitative PCR (AS-qPCR)

Primers were designed using a similar methodology used by the SNPgen online tool (http://snp.biotech.edu.lk/) [26] and custom synthesized by Macrogen Inc, Korea. To increase the specificity and feasibility of an efficient allelic discrimination, the following technique was used in primer designing: designing of primers for HRM and allele specific real time quantitative PCR (AS-qPCR) were based on a modified version of CADMA (competitive amplification of differentially melting amplicons) [27]. This method uses 3 primers–two allele specific primers and one common primer. Of the 2 allele specific primers, one primer is designed to amplify only the mutated allele (mutant primer) and the other primer amplifies only the wild-type allele (wild type primer). The common primer was designed to amplify both wild type and mutant alleles (Table 1). Furthermore, secondary mismatches were introduced in the allele specific primer sequences to increase the melting temperature (temperature increasing mutations—G or C mismatches) of one amplicon (either mutant or wild type amplicon) and temperature decreasing mutations (A or T mismatches) were introduced to decrease the melting temperature of the corresponding amplicon. Furthermore, to increase the specificity, a single mismatch was introduced at the second base from the 3´end of each allele specific primer.

**Table 1:** Primer sequences used for HRM and Allele specific real time quantitative PCR.

Gene	SNP	Primer Sequences (5’→3’)	Annealing temp	Method
FTO	Rs9939609	Forward wild type (T)—TAG GCT CCT CGC GAC TGC TGT GAA TAT T	6°C	HRM
		Forward mutant (A)—TAT GTT CAT TGC GAC TGC TGT GAA TAT A		
		Common reverse—GAG TAA CAG AGA CTA TCC AAG TGC ATC AC		
		Forward Seq Primer–CTG GCT CTT GAA TGA AAT AGG		
FSHB	Rs6169	Forward common primer- GTA CCT TCA AGG AAC TGG TAT	60°C	HRM
		Reverse wild type (C)—CGG GCA CTC TCA CTG TTC CG		
		Reverse mutant (T)—CAG GCA CTC TCA CTG TTA CA		
		Reverse Seq Primer–GCA CAG TAC AAT CAG TGC TGT CGC TGT C		
FSHR	rs6165 Thr307Ala	Forward wild type (T)–CAG AGA GAA TCT CTG AAC CCT AGT	60°C	HRM
		Forward mutant (C)–CAG AGA GGG TCT CTG AGC CCT AGC		
		Common reverse—GGC AAG AAG TTG ATT ATA TGA CTC AG		
		Forward Seq Primer–ACC CCA TGA TAT CTT CAC ATG GGT TGA A		
FSHR	rs6166 Asn680Ser	Forward wild type (T)–AGG GAC AAG TAT GTA AGT AGA ACC AT	60°C	HRM
		Forward mutant (C)—AGG GAC AAG TAT GTG AGT GGA ACC AC		
		Common reverse: CTC TTC AGC TCC CAG AGT CAC CA		
		Forward Seq Primer–CCA ATT TAC CTT AAA GGT ATG CCA		
INSR	Rs1799817	Forward common primer–ATG TCC CAC CCC CAC TGG ACT CAC AAC	60°C	HRM
		Reverse wild type (C)–TCG GTC ATG AAG GGC TTC ACC TGC CAT GAC		
		Reverse mutant (T)–TAA GTC ATG AAG GGC TTC ACC TGC CAT AAT		
		Reverse Seq Primer–CTC TGT GTA CGT GCC GGA CGA GTG GGA G		
GnRH	Rs6185	Forward common primer–TGG CTG GAG CAG CCT TCC ACT CA	68°C	AS-qPCR
		Reverse wild type (G)–CGC CTA GCT GGC CTT ATT CTA CTG ACG TG		
		Reverse mutant (C)–CTA CTA GCT GGC CTT ATT CTA CTG ACA TC		
		Reverse Seq primer–CTG ACT CTG ACT TCC ATC TTC TGC AGG G		
LHB	Rs1800447	Forward wild type (A)–ATT GCA TTG ATG GGG TGG CAA CA	63°C	AS-qPCR
		Forward mutant (G)–ATG GCA TTG ATG GGG TGG CAG CG		
		Reverse common primer–ATC CAG GGA GCC GCT TCG GAC A		
		Forward seq primer–CTG CCT CTG TGG GTC TGG CCC TGA GGT G		
LHB	Rs34349826	Forward common primer- AGC CCT CCT TCT CGA CAG CCT GG	68°C	AS-qPCR
		Reverse wild type (T)—CTA TGG TGC CAC CCC ATC AAT GCA AT		
		Reverse mutant (C)—CCG TGG TGC CAC CCC ATC AAT GCA AC		
		Reverse Seq primer- TCT TTG TGG GTG GTG TAC CAC GCG GGA		
LHCGR	Rs2293275	Forward wild type (T)–GTA TGC AAA TAC TTA CAG TGT TTT GTG AT	68°C	AS-qPCR
		Forward mutant (C)–AGC CGG CAA ATA CTT ACA GTG TTT TGT GAC		
		Reverse common primer–CAA TGT GAA AGC ACA GTA AGG AAA GTG A		
		Forward Seq primer–CAA TTG CAA AGA AAA AAT TCC CAT TTT A		

FTO—Fat mass and obesity-associated gene; FSHB—Follicle stimulating 225 hormone beta subunit; FSHR -Follicle-stimulating hormone receptor; INSR—Insulin receptor; GnRH—Gonadotropin-releasing hormone; LHB—Luteinizing hormone beta subunit; LHCGR -Luteinizing hormone/choriogonadotropin receptor. AS-qPCR–Allele specific quantitative PCR.

Since the HRM analysis works well for a melting temperature of around 60°C, SNPs with primers of melting temperature between 58–60°C were analyzed using the HRM method and primers with high melting temperature (above 60°C) analyzed using AS-qPCR. For the HRM analysis, all 3 primers (2 allele specific and 1 common primer) were added into one tube, whereas for allele specific PCR, each allele specific primer was added separately along with the common primer.

### PCR conditions

Real-time quantitative PCR was performed using a thermocycler (BioRad CFX 96). The final reaction mix for each assay consisted of 2 μl template DNA (10 ng/μl), 2 μl Solis BioDyne master mix (5x HOT FIREPol EvaGreen HRM Mix), 0.5 μl wild-type primer (10 μM), 0.5 μl mutation primer (10 μM), 0.5 μl common primer (10 μM), and finally adding 4.5 μl ddH_2_O to a final volume of 10 μl.

PCR cycling conditions and HRM conditions were: Initial denaturation 95°C for 15 minutes, followed by 40 cycles of denaturation (95°C for 15s,) annealing (60°C for 20s; annealing temperature varies with each SNP) and extension (72°C for 20s). HRM was performed from 50°C to 95°C with a temperature increase of 0.2°C/s with 50 acquisitions/°C.

Each sample was run in duplicate and each genotyping was carried out with a set of sequentially confirmed control samples of known genotypes (wild type, heterozygous and homozygous alleles).

**Fig 2:**
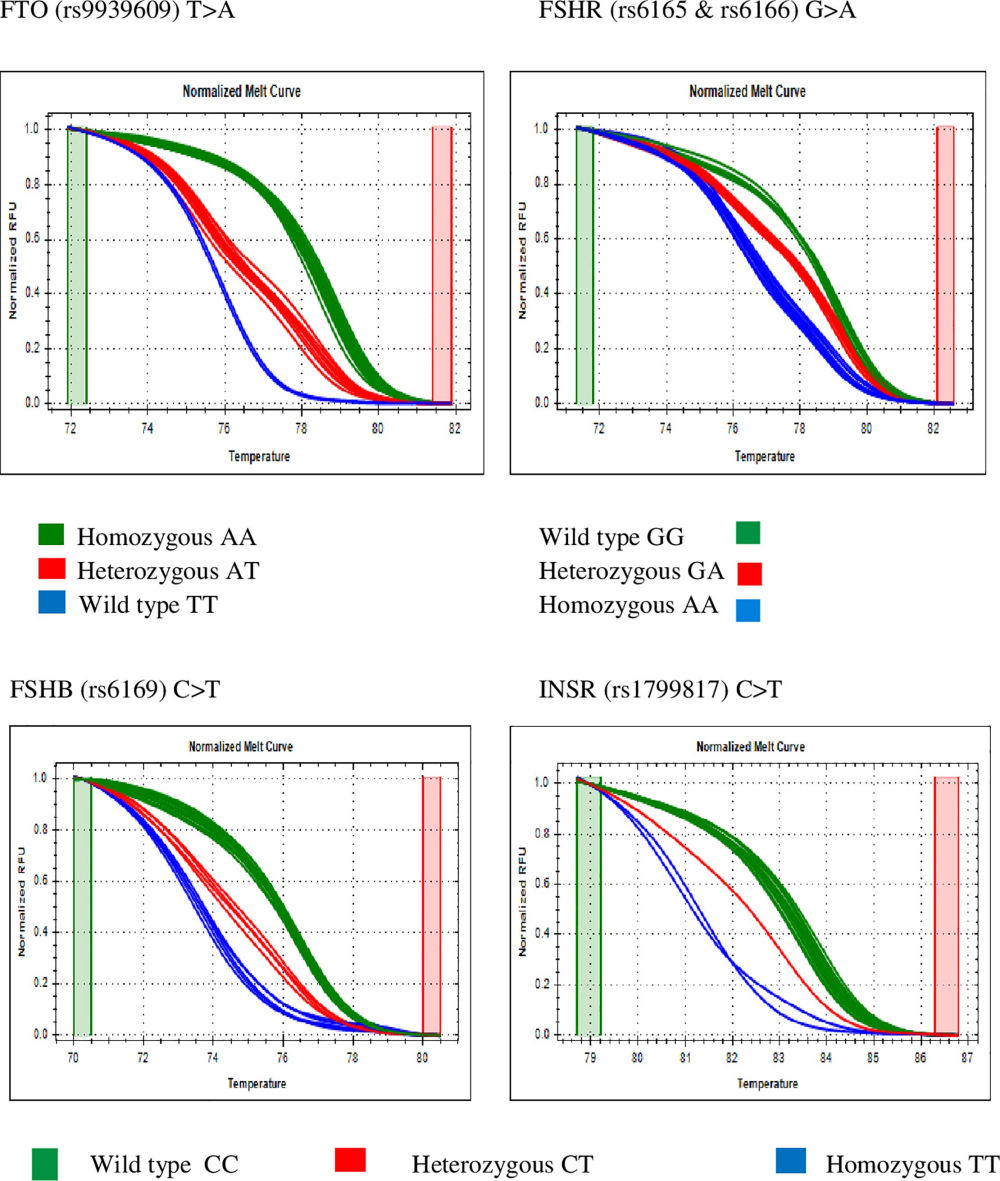
Normalized melt curve of HRM assay of SNPs–FTO (rs9939609), FSHR (rs6165/rs6166), FSHB (rs6169) and INSR (rs1799817).

Data was analyzed using Precision Melt Analysis software version 1.3. HRM assay melt curve results were normalized to identify the genotype (Fig 2). For allele-specific real time PCR, the amplification plot and respective Cq values were used for genotype identification.

The primer sequences and annealing temperature for each SNP is listed in Table 1.

### Sanger sequencing

Samples were randomly selected and the sequencing PCR was carried out on genomic DNA flanking the polymorphic site by using the sequence primer and the common primer from HRM or AS-qPCR and the amplified products were custom sequenced (Macrogen Inc, Korea) to validate the HRM and allele specific real time PCR methods. Primers used for sequencing are listed in Table 1.

### Tetra ARMS and Taq man assay for FTO genotyping

Additionally, tetra ARMS PCR and TaqMan assay (predesigned SNP genotyping assay, Thermo Fisher Scientific) were carried out for FTO (rs9939609) genotyping to confirm the HRM assay results. Tetra ARMS PCR primer details and PCR conditions are given in the supplementary material (S1 Table).

### Statistical analysis

Normal distribution of all clinical parameters was analyzed using the Kolmogorov-Smirnov test. Comparisons of normal distribution continuous variables between groups were analyzed using independent student’s t test. General clinical characteristics of cases and controls were expressed as mean ± standard deviation. The relative association between patients and controls for genotype and allele frequencies was assessed by Pearson’s χ2 test. The corresponding odds ratios (ORs) and confidence intervals (95% CIs) were calculated using SPSS version v.18.0 SPSS, Inc., Chicago, IL. The level of significance was set at 5%.

## Results

Demographic, clinical and hormonal characteristics of women with PCOS and controls are summarized in Table 2. Cases and controls showed no significant differences in relation to demographic characteristics, indicating the comparability of the two groups. The exception was the age difference between the two groups, which was close to being statistically significant (p = 0.06). This may have been due to chance, however it is unlikely to have affected the risk, as age is usually not associated as a confounder with genes tested. Women with PCOS had significantly higher BMI and mFG score. Serum kisspeptin and testosterone concentrations were significantly higher in women with PCOS (kisspeptin– 4.873 nmol/L; testosterone—4.713 nmol/L) than controls (kisspeptin– 4.127 nmol/L; testosterone—3.415 nmol/L p<0.05).

**Table 2:** Demographic, clinical and biochemical characteristics of the study population.

	PCOS (n = 55)	CONTROLS (n = 110)	p
Age (Years)	24.67 ± 0.883	33.80 ± 0.528	0.061
BMI(Kg/m^2^)	26.89 ± 0.716	25.25 ± 0.344	0.007
mFG score	8 ± 0.445	3 ± 0.222	0.006
WC:HC	0.839 ± 0.008	0.824 ± 0.004	0.114
FBG (mg/dL)	98.81 ± 2.08	108.69 ± 2.74	0.284
Kisspeptin (nmol/L)	4.873 ± 0.238	4.127 ± 0.132	0.033
Testosterone (nmol/L)	4.713 ± 0.458	3.415 ± 0.256	0.018
TSH (μIU/mL)	1.96 ± 0.346		
FSH (mIU/mL)	5.5 ± 0.430		
LH (mIU/mL	7.34 ± 1.198		

BMI–Body mass index; mFG–modified Ferriman-Gallway score; WC:HC–waist circumference: hip circumference; FBG–fasting blood glucose; TSH–Thyroid stimulating hormone; FSH–Follicle stimulating hormone; LH—Luteinizing hormone.

### Validation of HRM assay and allele-specific real-time quantitative PCR

The FTO, FSHR, FSHB and INSR genes were genotyped using HRM method (Fig 2) and GnRH1, LHB and LHCGR genes were genotyped using AS-qPCR method. Samples were randomly selected and validated by Sanger sequencing.

**Fig 3:**
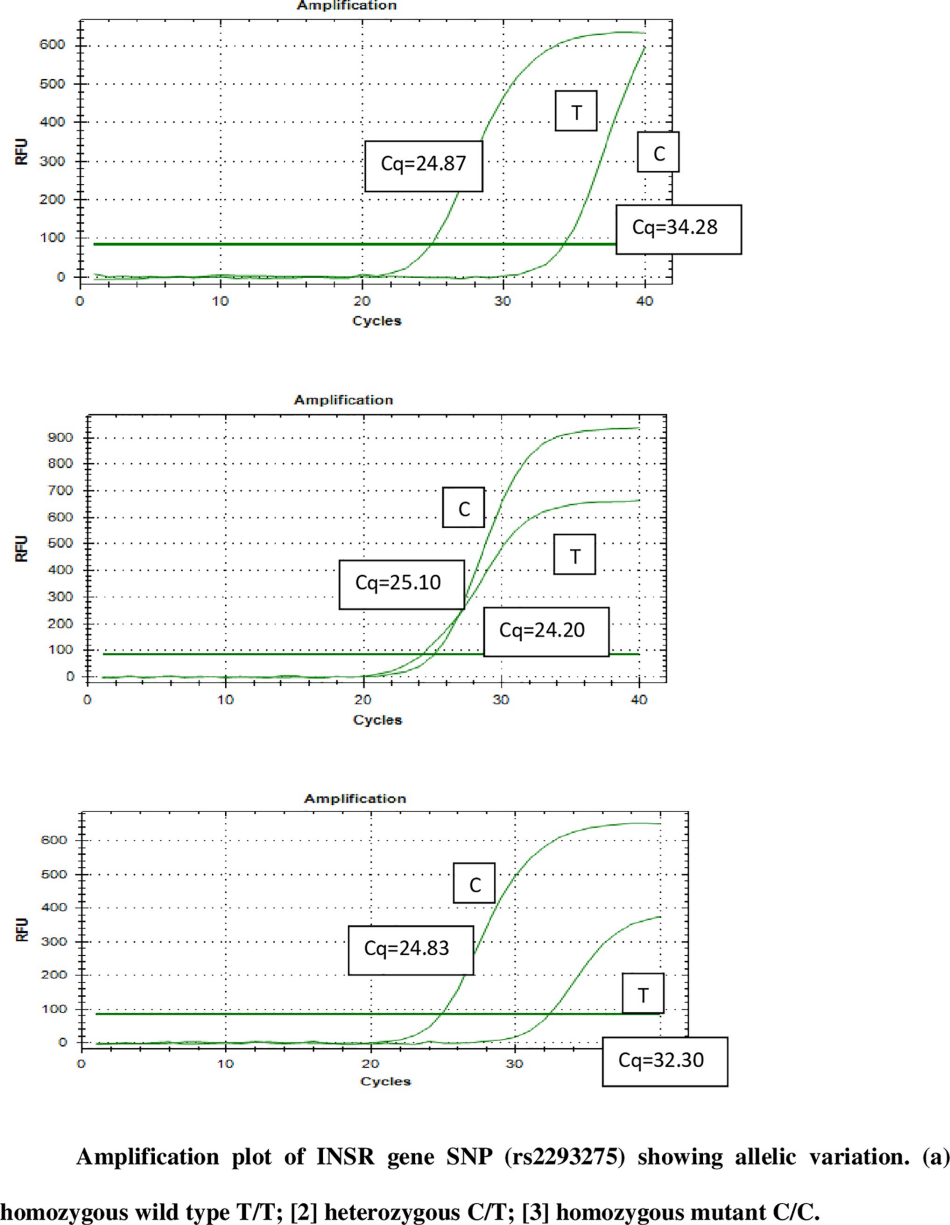
Amplification plot of INSR gene SNP (rs2293275).

Fig 3 illustrates the amplification plot of SNP–rs2293275 (INSR). Cq values <30 indicates positive amplification of the target. If the Cq value of one primer set is > 30, it indicates the absence of the respective allele in the sample; thereby homozygous towards the other allele (S2 Fig). When both primer sets show Cq value < 30, the sample is heterozygous. The allele specificity of Cq values <30 was decided based on several trials with different human DNA samples for all SNPs and validated by Sanger sequencing.

Comparing the results of the two genotyping methods (HRM and AS-qPCR), there was 100% accordance between the results of these two assays and the sequencing results. In samples evaluation all duplicates had similar results, indicating consistency and reproducibility of the assay.

### Genotype frequencies

Genotype distribution of all the studied polymorphisms of 55 women with PCOS and 110 ethnically matched controls are shown in Table 3.

**Table 3:** Genotypes distribution in cases and controls.

Gene	Genotype	Cases (n = 55)	Controls (n = 110)	P	OR (95% CI)
FTO	T/A (rs9939609)				
	TT	20 (36.4%)	72 (65.4%)		1^a^
	AT	13 (23.6%)	23 (20.9%)	0.95	2.035(0.877–4.720)
	AA	22 (40%)	15 (13.6%)	0.0001	5.28 (2.320–12.016)
	AT+AA	35 (63.6%)	38 (34.5%)	0.0001	3.316 (1.687–6.515)
GnRHI	G/C (rs6185)				
	GG	50 (90.9%)	96 (87.3%)		1^a^
	GC	05 (9.1%)	14 (12.7%)	0.490	0.686 (0.234–2.013)
	CC	00	00		
FSHB	C/T (rs6169)				
	CC	37 (67.3%)	66 (60%)		1^a^
	CT	02 (3.64%)	03 (2.73%)	0.853	1.189 (0.190–7.442)
	TT	16 (29.1%)	41 (37.3%)	0.312	0.696 (0.344–1.408)
	CT+TT	18 (32.7%)	44 (40%)	0.363	0.730 (0.370–1.441)
FSHR	G/A (rs6165 & rs6166)				
	GG	13 (23.6%)	29 (26.4%)		1^a^
	GA	26 (47.3%)	53 (48.2%)	0.826	1.094 (0.489–2.448)
	AA	16 (29.1%)	28 (25.4%)	0.596	1.275 (0.520–3.127)
	GA+AA	42 (76.4%)	81 (73.6%)	0.705	1.157 (0.545–2.456)
LHB	T/C (rs 1800447)				
	TT	00	00	-	-
	CT	00	00	-	-
	CC	55 (100%)	110 (100%)	-	-
	AG (rs34349826)				
	AA	00	00	-	-
	AG	00	00	-	-
	GG	55 (100%)	110 (100%)	-	-
LHCGR	A/G (rs2293275)				
	AA	11 (20%)	29 (26.4%)		1^a^
	GA	31 (56.4%)	44 (40%)	0.142	1.857 (0.808–4.270)
	GG	13 (23.6%)	37 (33.6%)	0.873	0.926 (0.362–2.368)
	GA+GG	44 (80%)	81 (73.6%)	0.369	1.432 (0.653–3.140)
INSR	C/T (rs1799817)				
	CC	48 (87.3%)	95 (86.4%)		1^a^
	CT	01(1.82%)	01 (0.9%)	0.626	1.979 (0.121–32.33)
	TT	06 (10.9%)	14 (12.7%)	0.751	0.848 (0.307–2.346)
	CT+TT	07 (12.7%)	15 (13.6%)	0.871	0.924 (0.353–2.417)

OR–odds ratio; CI–confidence intervals; FTO—Fat mass and obesity-associated gene; FSHB—Follicle stimulating hormone beta subunit; FSHR -Follicle-stimulating hormone receptor; INSR—Insulin receptor; GnRH—Gonadotropin-releasing hormone; LHB—Luteinizing hormone beta subunit; LHCGR -Luteinizing hormone/choriogonadotropin receptor.

^a^ Reference genotype

The genotype frequencies of the SNPs GnRH1 (rs6185), FSHB (rs6169), FSHR (rs6165 & rs6166), LHB (rs1800447 & rs34349826), LHCGR (rs2293275) and INSR (rs1799817) were not significantly different between cases and controls (p>0.05). However, in the FTO gene rs9939609 polymorphism, a higher frequency of A allele (mutant allele) was observed in the PCOS group, while the frequency of T allele (normal allele) was significantly higher in the control group (Cases—AA = 40%, AT = 23.6%, TT = 36.4%; Controls—AA = 13.6%, AT = 20.9%, TT = 65.4%; p<0.05). AA genotype was positively associated with PCOS in our sample (OR = 5.28; 95% CI = 2.320–12.016; p<0.05). A significant correlation was also found between FTO gene and BMI (chi square value = 17.05, p<0.05).

Meanwhile, GnRH1 (rs6185) homozygous mutant CC genotype was not detected among cases or controls. In terms of FSHR (rs6165 and rs6166) and LHCGR (rs2293275) polymorphisms, heterozygous genotype (GA) was more frequently observed in the population studied. Although no statistically significant difference was observed in genotype distribution between cases and controls in rs2293275 (LHCGR) polymorphism (p>0.05), mutant homozygotes (GG) were observed at a higher frequency than the normal wild type (AA) genotypes in both groups. Moreover, in rs1799817 (INSR) polymorphism, TT and CT genotype frequencies were found to be considerably less in cases and controls than those of their wild type genotype (CC). Furthermore, only homozygous mutant genotypes, CC and GG were present in both cases and controls in LHB gene polymorphism rs1800447 and rs34349826 respectively (Table 3). The latter two polymorphisms are in linkage disequilibrium (rs1800447/rs34349826), while the wild type and heterozygous genotypes were not detected.

## Discussion

There is a growing demand for rapid and reliable genotyping in chronic conditions of unclear aetiology, such as PCOS, and in particular among differing ethnic groups. In recent years, conventional polymerase chain reaction (PCR) techniques have been replaced by quantitative real-time PCR (qPCR). The benefits of qPCR in relation to conventional PCR include speed, reproducibility and quantitative ability. Additional operational advantages of qPCR include greater sensitivity and reproducibility, with the potential to replace conventional PCR in routine diagnostic practice. It is noteworthy that HRM and AS-qPCR have been used by some lead centers for genotyping [27–30]. To the best of our knowledge this is the first report on SNPs of selected genes associated with PCOS among Sri Lankan women and also the first study to use HRM and AS-qPCR for genotyping.

We acknowledge that cases and control were not strictly age matched, where the mean age of controls is higher than that of PCOS subjects (p = 0.06). Our explanation for this marginally older cohort of controls is that we sought volunteers from a large work setting; when we had to ensure they were truly normal with regular cycles over a period of time, had achieved fertility and clearly did not express even the milder phenotypes of PCOS. We propose that this age difference is unlikely to impact on their genetic makeup and thereby the findings of this study.

The HRM method uses mutation introduced primers that produce amplicons which differ in nucleotide content based on the genotype. The nucleotide content is reflected in the melting properties of the amplicon, detectable through HRM analysis. Furthermore, a mismatch introduced in the two allele specific primers at the second base from the 3´terminus, increases the specificity of PCR amplification and weakens non-specific amplifications and thereby limits false amplification. In addition, by increasing the melting temperature of one allele specific primer and decreasing the melting temperature of another allele specific primer, the HRM method allows sufficient difference in the melt curve to distinguish between homozygous and heterozygous alleles.

Recent genome-wide association studies reported that FTO gene variants are associated with PCOS, mostly in Asians [31]. We found the FTO gene rs9939609 polymorphism to be associated with PCOS in Sri Lankan women (OR = 3.316; 95% CI = 1.687–6.515; p<0.05). Furthermore, we repeated the FTO genotyping using Tetra ARMS PCR and TaqMan assay to confirm our findings, which mirrored the results by HRM method (S1 Fig). Table 4 compares results of this study with other reports. Barber et al. [32], Yan et al. [33], Sokkary et al. [34] and Farhan et al. [35] reported an association between the FTO rs9939609 variant and PCOS, which mirror our findings. Meanwhile, Tan et al. [36], Wehr et al. [37] and Ramos et al. [38] showed no association between SNPs in FTO and the PCOS phenotype.

**Table 4:** Comparison of genotype associations with PCOS–current study versus. other reports.

Gene	SNP	Mutation	A.A change	Association in present study (Yes/ No)	Association in other studies (Yes/ No)	Population
FTO	Rs9939609	T/A	Intron	Yes	Yes	UK [32]
					Yes	Chinese [33]
					Yes	Egypt [34]
					Yes	Iraq [35]
					No	Germany [36]
					No	Austria [37]
					No	South Brazil [38]
GnRH1	Rs6185	G/C	Trp → Ser	No	No	Western European descent [39]
FSHB	Rs6169	C/T	Tyr → Tyr	No	No	Singapore Chinese [40]
FSHR	Rs6165	G/A	Ala → Thr	No	No	Korean [43]
					No	Turkey [44]
					No	Singapore Chinese [46]
					No	Han Chinese [47]
					Yes	Italian [41]
					Yes	Chinese [42]
	Rs6166	G/A	Ser → Asn	No	No	Turkey [44]
					No	UK [45]
					No	Singapore Chinese [46]
					No	Han Chinese [47]
					Yes	Korean [43]
LHB	rs1800447/ rs3449826	T/C A/G	Trp → Arg IIe → Thr	No	Associated with ↑ Testosterone levels	Brazil [51]
LHCGR	Rs2293275	A/G	Asn → Ser	No	No	Western European descent [39]
					Yes	Sardinian [52]
					Yes	Hui Chinese [53]
					Yes	Egypt [54]
INSR	Rs1799817	C/T	His → His	No	No	Korean [55]
					Yes-lean patient	Indian [24]

FTO—Fat mass and obesity-associated gene; FSHB—Follicle stimulating 348 hormone beta subunit; FSHR—Follicle-stimulating hormone receptor; INSR—Insulin receptor; GnRH—Gonadotropin-releasing hormone; LHB—Luteinizing hormone beta subunit; LHCGR -Luteinizing hormone/choriogonadotropin receptor.

In the current study, GnRH1 (rs6185) gene polymorphism had an approximately equal distribution of wild type (GG) and heterozygous (GC) alleles between PCOS and controls subjects; while the homozygous mutant allele (CC) was not present in cases and controls. Meanwhile, FSHB gene polymorphism (rs6169) showed a similar distribution of wild type (CC) and heterozygous (CT) alleles in PCOS and controls subjects. Nonetheless, the frequency of FSHB gene homozygous mutant (TT) allele was 37.3% among the control subjects compared to 29.1% in patients. We also found no significant association between GnRH1 and FSHB gene polymorphisms with PCOS (Table 3) (p>0.05). The few studies reported (Table 4) to identify any association of rs6185 (GnRH1) and rs6169 (FSHB) polymorphisms with PCOS, also failed to find any association [39, 40]. Therefore, we conclude that GnRH1 (rs6185) and FSHB (rs6169) gene mutations are uncommon in Sri Lankan subjects with well characterized PCOS manifesting from adolescence.

We also observed that the polymorphisms of FSHR rs6165 and rs6166 had no significant association with PCOS. A few researchers [41, 42] have reported an association between FSHR gene polymorphisms (rs6165 and rs6166) and PCOS, while the majority failed to find any association [43–47] (Table 4). Meanwhile, Valkenburg et al. [39] concluded that FSHR gene variants were strongly associated with the severity of PCOS and its clinical features, but not with the disease risk.

Studies have identified two common mutations in the LHB gene associated with PCOS; one in codon 8 (rs1800447) and other in codon 15 (rs34349826) and that these two polymorphisms (rs1800447 & rs34349826) exist in complete linkage disequilibrium [48]. Meanwhile, some have shown this LHB gene variant to represent a universal polymorphism [48, 49]. However, the LHB gene variant has been reported to be so far less common in Asians [49, 50]. Conversely, Batista et al. [51] showed that LHB gene polymorphisms (rs1800447/rs34349826) were associated with elevated testosterone levels in women with PCOS. In our analysis, LHB gene polymorphisms (rs1800447 and rs34349826) were detected only as homozygous mutant genotypes (CC and GG respectively) in both PCOS and control subjects. It is noteworthy that in the rs1800447 polymorphism, the point mutation in codon 8 causes amino acid replacement from Trp to Arg; while in rs34349826 polymorphism the point mutation in codon 15 causes amino acid replacement from Ile to Thr [48]. The resulting alterations of the amino acids, Trp (hydrophobic) to Arg (hydrophilic) and Ile (hydrophobic) to Thr (hydrophilic) may themselves introduce significant conformational changes to the LH protein. Since our study population have only mutated genotypes of LHB gene polymorphisms (rs1800447 and rs34349826), it can be argued that these variant LH proteins have little or no impact on the phenotype of PCOS in our study population. This study also suggests that the LHCGR polymorphism (rs2293275) is most unlikely to be associated with the pathogenesis of PCOS. Conversely, the majority of studies [52–54] indicate that rs2293275 polymorphism in exon 10 of the LHCGR gene variant is strongly linked with PCOS (Table 4), while there are only a few reports that rs2293275 polymorphism is not associated with PCOS [39].

We did not find significant differences in the genotype distribution between cases and controls of rs1799817 polymorphism of the INSR gene (p>0.05). This is consistent with findings of Lee et al. [55], where no association was found between rs1799817 polymorphism and PCOS susceptibility in a Korean population [55]. On the other hand, an Indian study showed polymorphism of INSR gene as a susceptibility factor in patients with PCOS, especially in non-obese PCOS patients [24]. Yet, another Indian study reported the INSR gene SNP (rs1799817) was associated with increased insulin resistance in Indian women with PCOS [25]. However, we did not find any relationship between clinical and biochemical characteristics and the INSR gene polymorphism (rs1799817) among women with PCOS (p>0.05). Such discrepancies in results may be due to variations in study design, sampling technique and sample size, along with demographic and genetic differences among study populations.

Most importantly, we have found that serum kisspeptin levels were significantly higher in adult subjects with PCOS having the well characterized phenotype from adolescence than ethnically matched controls (Table 2). In addition, our study of the Kiss 1 and the GPR54 receptor genes revealed 2 and 5 SNPs respectively (manuscript in preparation), although these SNPs were not associated with PCOS.

Therefore, in summary, it can be assumed that selected SNPs of HPG axis genes are not likely to be associated with PCOS in Sri Lankan women. These findings, no doubt require validation by further large scaled studies.

## Conclusions

This is the first study of Sri Lankan women with well characterized PCOS carried out to explore any association with multiple SNPs of different pathophysiological pathways implicated in the aetiology of PCOS. The rs9939609 variant of FTO gene is significantly associated with PCOS among Sri Lankan women, reflecting its effect on central adiposity. Meanwhile, the gene polymorphisms representing the HPG axis (GnRH1, FSHB, FSHR, LHB and LHCGR) and INSR gene polymorphism do not show any significant association with PCOS. Interestingly, only homozygous mutant genotypes were present in LHB gene polymorphism (rs1800447 and rs34349826) in both PCOS and control subjects.

Moreover, HRM and allele specific real time PCR are simple, fast, cost-effective and efficient genotyping techniques, feasible in many diagnostic units, as real time PCR instruments are standard equipment in most molecular diagnostic laboratories. The limitation of these two assays is that initially samples need to be validated by sequencing method to confirm the results. However, by applying samples of known genotypes as a reference, the two assays can be used for reliable genotyping of samples of unknown status.

## Supporting information

S1 File Genotype results of the study population. Genotype data of the cases and controls.
(XLSX)

S2 File Demographic and clinical characteristic of study population. Demographic, clinical and hormonal data of the cases and controls.
(XLSX)

S1 Fig Genotyping of FTO (rs9939609) polymorphism by TaqMan assay and Tetra ARMS-PCR. Allelic discrimination plot of FTO (rs9939609) polymorphism by TaqMan assay and detection of rs9939609 gene polymorphism by Tetra ARMS-PCR (PCR products on 2% agarose gel).
(DOCX)

S2 Fig Genotyping of GnRH1 and LHB polymorphism by AS-qPCR. Amplification plot of GnRH1 and LHB genes single nucleotide polymorphisms.
(DOCX)

S1 Table Primer details and PCR conditions for Tetra ARMS PCR of FTO rs9939609 polymorphism. (DOCX)
