# Supplementary material for: Identification of selected genetic polymorphisms in polycystic ovary syndrome in Sri Lankan women using low cost genotyping techniques
Source: PLoS One. 2018 Dec 31;13(12):e0209830. doi: 10.1371/journal.pone.0209830 (PMC6312267; doi:10.1371/journal.pone.0209830)
Supplement: S1 Fig — Allelic discrimination plot of FTO (rs9939609) polymorphism by TaqMan assay and detection of rs9939609 gene polymorphism by Tetra ARMS-PCR (PCR products on 2% agarose gel). (DOCX) [file pone.0209830.s003.docx]

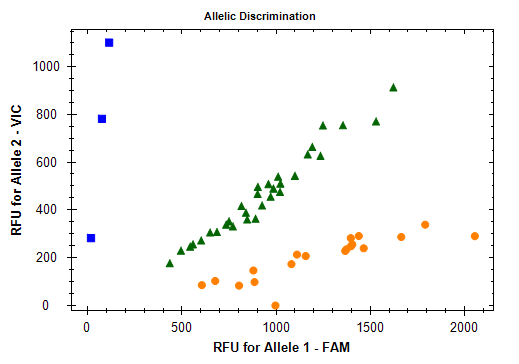


Allele 1 Both allele (Heterozygous) Allele 2

**Fig 1: Allelic discrimination plot of FTO (rs9939609) polymorphism by TaqMan assay**

**M 1 2 3 4 5 6 7 8**


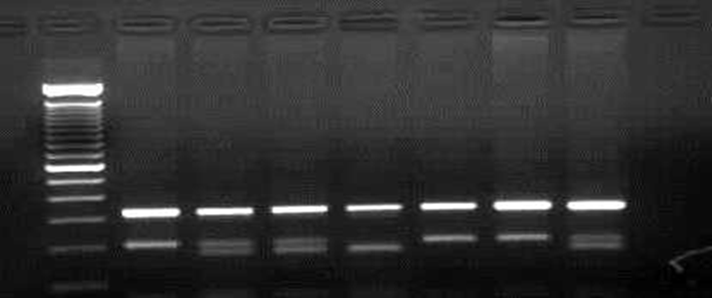


321bp

201bp

178bp

**Fig 2: Detection of rs9939609 gene polymorphism by Tetra ARMS-PCR. PCR products on 2% agarose gel. M: 100bp ladder, Lanes 1,5,6: AA genotype, lanes 2,3 7: AT genotype, lane 4: TT genotype and lane 8: negative control.**
