# Supplementary material for: Identification of selected genetic polymorphisms in polycystic ovary syndrome in Sri Lankan women using low cost genotyping techniques
Source: PLoS One. 2018 Dec 31;13(12):e0209830. doi: 10.1371/journal.pone.0209830 (PMC6312267; doi:10.1371/journal.pone.0209830)
Supplement: S2 Fig — Amplification plot of GnRH1 and LHB genes single nucleotide polymorphisms. (DOCX) [file pone.0209830.s004.docx]

**GnRH1 (rs6185)**


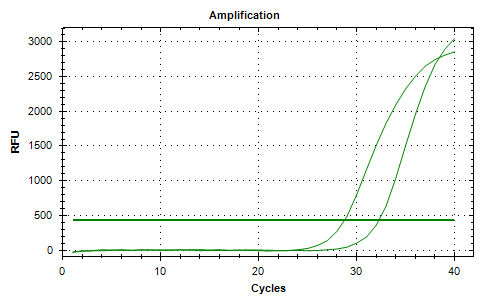


Cq=32.26

Cq=28.74

**Fig 1: Amplification plot of GnRH1 gene SNP - rs6185 polymorphism - wild type (GG)**

**LHB (rs1800447 / rs34349826)**


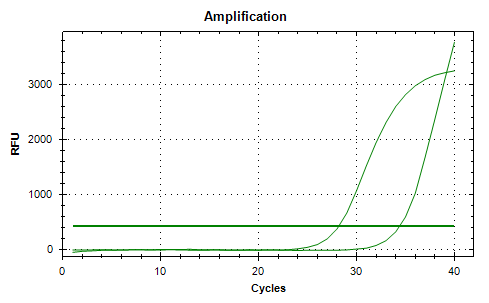


Cq=34.37

Cq=28.17

**Fig 2: Amplification plot of LHB gene SNP - rs1800447/rs34349826 polymorphism – homozygous mutant (CC/GG)**
