# Supplementary material for: Identification of selected genetic polymorphisms in polycystic ovary syndrome in Sri Lankan women using low cost genotyping techniques
Source: PLoS One. 2018 Dec 31;13(12):e0209830. doi: 10.1371/journal.pone.0209830 (PMC6312267; doi:10.1371/journal.pone.0209830)
Supplement: S1 Table — (DOCX) [file pone.0209830.s005.docx]

| **Gene** | **SNP** | **Primer Sequences (5’→3’)** |
| --- | --- | --- |
| FTO | Rs9939609 | F_out_ - 5'-TGG CTC TTG AAT GAA ATA GGA TTC AGA A |
|  |  | R_out_ (A) - AGC CTC TCT ACC ATC TTA TGT CCA AAC A |
|  |  | F_in_ - TAG GTT CCT TGC GAC TGC TGT GAA TAT A |
|  |  | R_in_ - GAG TAA CAG AGA CTATCC AAG TGC ATC TCA |

**Table 1: Primer details for Tetra ARMS PCR**

**Table 2: PCR conditions for Tetra ARMS PCR**

| **Step** | **Temperature** | **Duration** |
| --- | --- | --- |
| Initial denaturation | 94°C | 5 minutes |
| Denaturation | 94°C | 30 sec |
| Annealing | 53°C | 25 sec |
| Extension | 72°C | 25 sec |
| Final extension | 72°C | 10 minutes |

40 cycles
